# Occurrence of butyrylcholinesterase polymorphisms in patients undergoing surgery in Slovakia

**DOI:** 10.22088/cjim.14.3.490

**Published:** 2023

**Authors:** Lukáš Čuchráč, Marta MydlárováBlaščáková, Jozef Firment, Jana Šimonová, Bernasovská Jarmila, Janka Vašková

**Affiliations:** 1 1st Clinic of Anaesthesiology and Intensive Care Medicine, Louis Pasteur University Hospital in Košice, and PavolJozefŠafárik University in Košice, Tr. SNP 1, 040 66 Košice, Slovak Republic; 2 Department of Biology, Faculty of Humanities and Natural Sciences, University of Prešov in Prešov, Ul. 17 novembra 1, 081 16 Prešov, Slovak Republic; 3 Department of Medical and Clinical Biochemistry, Faculty of Medicine, PavolJozefŠafárik University in Košice, Tr. SNP 1, 040 66 Košice, Slovak Republic

**Keywords:** Butyrylcholinesterase, Cholinesterase, Neuromuscular blocker, Polymorphism, Residual curarization

## Abstract

**Background:**

Post-operative residual curarization is a persisting problem, characterized by muscle fatigue, exhaustion or paresis, caused by the use of neuromuscular blocking agents with prolonged postoperative effect. Genetically, determined changes in cholinesterase activity can be a major reason for persistent muscle blockade after administration of muscle relaxants.

**Methods:**

Regarding the subsistence of polymorphisms in the plasma cholinesterase gene causing change in enzyme activity and metabolism of applied drugs, we investigated the frequency of two polymorphisms known to reduce its activity significantly in patients undergoing surgery.

**Results:**

Primary results show a relatively high occurrence of plasma cholinesterase K risk allele (18.75%).

**Conclusion:**

Characterization of the lacking information about genetic background of changes in plasma cholinesterase activity within Slovakia may allow for easier decision-making in clinical practice when selecting alternative neuromuscular blocking and also reversal agents.

Neuromuscular blocking agents (NMBAs) are part of everyday anaesthesia practice around the world. An estimated 51.4 million surgical procedures are carried out in the USA alone (1), with an estimated 34.8 million procedures in Europe (2) and a suggested combined global estimate of 234.4 million surgeries per year. Post-operative residual curarization (PORC) is characterized by the presence of muscle fatigue, exhaustion or paresis due to the use of NMBAs with prolonged postoperative effect. PORC is connected with hypoxia, muscle flaccidity causing respiratory failure, thus increasing morbidity during all phases of surgical procedure. These symptoms, such as weakness, hypoxia or an inability to cough, can occur relatively frequently, but are rarely associated with residual curarization. Studies have shown an incidence of PORC ranging from 4 - 50% relying on diagnostics, the NMBA used, and the application of the drugs to reverse muscle paralysis (3, 4). 

Murphy and Brull (5) also report an incidence ranging from 2% to 64%. This problem has significant clinical consequences such as muscle weakness, decreased saturation, alveolar disruption, and acute respiratory failure, leading to serious central nervous system damage and even death. Succinylcholine (and rocuronium) are typically used as NMBAs when neuromuscular blockade is required to rapidly facilitate tracheal intubation (6). It is hydrolysed by plasma cholinesterase (butyrylcholinesterase, BCHE, pseudocholinesterase). Normally, the reestablishment of neuromuscular activity is approximately within 9 minutes, albeit with significant inconstancy.

This may be based either on attained or hereditary aspects (7). BCHE does not metabolize rocuronium, and sugammadex can be used to reverse persistent muscle block (8). However, rocuronium is not recommended for safe use in all patients due to not same quick action as succinylcholine, hepatotoxicity association (9) and adverse consequences for neonates when used for general anaesthesia in obstetrics (10). 

In addition, elimination of the block by sugammadex is not always successful in patients with myasthenia gravis (11). BCHE deficiency has an autosomal recessive inheritance, with many variants that can cause some degree of reduced choline ester metabolism. Despite this, it does not pose any additional health risks, so can only be detected after the use of succinylcholine or mivacurium. In about 25% of Caucasians occur mutations in the BCHE gene (12, 13). Majority of the variants are single nucleotide polymorphisms (14). Polymorphism rs1803274 is associated with an increased risk of coronary restenosis (15), Alzheimer's disease (16), tumorigenesis (17) and metabolic risk factors (cardiovascular, obesity, blood pressure, and diabetes) (18).This study was aimed to assess the frequency of *BCHE *polymorphisms, responsible for reduced BCHE activity in patients undergoing surgery in Slovakia.

## Methods

The Ethics Committee of Louis Pasteur University Hospital in Košice approved the study under no. 115/EC/18. Participants (32) underwent surgery under general anaesthesia using a non-depolarizing relaxant – rocuronium with an intermediate duration of action. All participants were extubated in the operating room and, accompanied by an anaesthesiologist and anaesthesiology nurse, transported to a recovery room, where they were immediately connected to a vital signs monitor. 

Oxygen was applied via facemask as part of oxygen therapy and a TOF-Watch muscle relaxation monitor (Organon, SwordsCo., Dublin, Ireland) was attached to their forearms. The ulnar nerve was stimulated by TOF stimulation (4 pulses lasting 0.2 ms and with a frequency of 2 Hz).

 Three consecutive TOF measurements were taken 15 minutes apart and averaged. Patients were released from the recovery room only after reaching TOF ratio values ≥ 0.9. Weight, height (BMI calculated), gender, age, duration of exercise, body temperature and American Society of Anaesthesiologist physical status classification (ASA) were also recorded. Information on patient decurarization and sugammadex administration was also recorded in the protocol. Clinical conditions of patients with low levels of TOF ratio were monitored for longer period and they were released from the recovery room after reaching TOF values ≥ 0.9. 

Molecular genetic analysis was performed from DNA isolated from patients' venous blood after collection into tubes with K_3_EDTA. DNA isolation was provided by UltraClean®DNA Isolation Kit (non-spin) (MoBio, CA, USA). BCHE gene polymorphisms (rs1799807, rs1803274) with reduced butyrylcholinesterase activity up to 30% were selected. Genotyping was realized using the TaqMan SNP Assay (C___2411904_20; C__27479669_20) (Applied Biosystems, CA, USA) according to standard protocol followed by Real-Time PCR fluorescence detection using a 7500 Fast Real-Time PCR System (Applied Biosystem). The genotype with mutation was verified by sequencing and used as a positive control for other analysis. The resulting genotypes were subtracted from the allelic discrimination graph and the amplification curves of the individual alleles.

## Results

We provided initial analysis of genetic background of two single nucleotide polymorphisms responsible for approximately 30% reduced activity of BCHE. From 32 individuals, only 2 were carriers of risk allele A for rs1799807 polymorphism and were at risk with succinylcholine use with respect to the heterozygous genotype (table 1). 

Prevalent genotype posing no risk was found in 30 patients undergoing surgery.For the second polymorphism, rs1803274, the incidence of the risk allele A (so called K variant) was relatively high. In the small sample examined so far 12 (18.75%) patients and 10 (31.25%) patients carried the risk genotype. One of the examined patients was homozygous. Combinations of the most common genotypes have not yet confirmed the coexistence of both variants in homozygous individuals. Two patients carried risk alleles for both variants (table 2). When comparing with need for medications used for rocuronium neuromuscular block reversal, three patients carrying K variant were used to end blockade by sugammadex. However, five variant K carriers were treated by neostigmine.

**Table 1:** Allele and genotype frequencies of the rs1799807, rs1803274 polymorphisms of the BCHE gene in patients undergoing surgery

**Polymorphism**	**Allele** **frequencies**		**Genotype** **frequencies**		
	**G**	**A**	**GG**	**GA**	**AA**
**rs1799807**	2	62	-	2	30
	3.12%	96.88%	0.00%	6.25%	93.75%
**rs1803274**	52	12	21	10	1
	81.25%	18.75%	65.63%	31.25%	3.12%

**Table 2:** Combined genotypes in patients undergoing surgery

**Polymorphism**		**Occurrence**	
**rs1799807**	**rs1803274**	**N**	**%**
GG	GG	-	-
GG	GA	-	-
GG	AA	-	-
GA	GG	-	-
**G**A	G**A**	2	6.25
GA	AA	-	-
AA	GG	21	65.63
AA	G**A**	8	25.00
AA	**AA**	1	3.12

## Discussion

Genetic analyses of BCHE polymorphisms to diagnosticate patients at risk of prolonged post-succinylcholine neuromuscular blockade concede easier decision-making in clinical practice when selecting alternative NMBAs. Up to date, at least 75 genetic polymorphisms are known for BCHE (19). Genetic variants cause a lack of BCHE activity, preserving higher concentrations of succinylcholine, and persisting neuromuscular blockade. Patients carrying variants are usually asymptomatic until administered succinylcholine during surgery (20). The known variant of BCHE is the atypical variant (variant "A"). The atypical variant is based on the single nucleotide substitution polymorphism rs1799807 (G), which results in the exchange of aspartate for glycine at the secondary substrate/inhibitor binding site. The "A" form of BCHE has 30% lower enzyme activity (21). Presence of the atypical variant is the most common cause of prolonged apnoea (22) and use of succinylcholine is not recommended for patients with a homozygous genotype for this variant. In our initial analysis of 32 samples, only two individuals were carriers, e.g. at risk with succinylcholine use with respect to the heterozygous genotype (table 1).

The A allele of the second analyzed single nucleotide polymorphism rs1803274 encodes a variant of Kalow (K) caused by the exchange of guanine for adenine at nucleotide 1615, leading to the exchange of the amino acid alanine for threonine at codon 539 (Ala539Thr). As a result, BCHE activity is reduced and leads to a 30% prolongation of the duration of neuromuscular blockade even after administration of mivacurium (23,24). Results from our study showed the incidence of the risk allele for the rs1803274 polymorphism was relatively high in the small sample examined so far (18.75%) and almost 31.25% of individuals carried the risk genotype. One of patients was even homozygous for variant K. So far, it is a very interesting finding that combinations of the most common genotypes have not yet confirmed the coexistence of both variants in homozygous individuals. The importance of the variant K for the persistence of succinylcholine effect has not been well studied, although a frequency of alleles reached up to 0.27 in some populations native to North America (12,24), Japan (25), Brazil (26), Italy (27), Denmark (28); in all, approximately 1 in 63 individuals carried a homozygous genotype (24). This seems to be less common than in our study.

Non-depolarizing relaxants act by competitive inhibition of acetylcholine receptors. For example, atracurium is not metabolized by BCHE but by ester bond hydrolysis or Hoffman elimination. Vecuronium and rocuronium are excreted in the bile (29). Only rocuronium, which is not metabolised by BCHE, was used in the study. In three (16.6%) patients, it was necessary to reverse the effect of rocuronium with sugammadex, which forms complex with it or a neostigmine that inhibits acetylcholine hydrolysis by competing for cholinergic transmission sites on acetylcholinesterase. Atypical BCHE is resistant to inhibition by neostigmine or physostigmine. Even up to 20-fold higher concentrations lead to 50% inhibition of atypical BCHE (30). However, five neostigmine decurarised patients were carriers of variant K. polymorphisms expressing abnormally low enzyme concentrations, inclusive of variant K and silent variants, are especially responsive to low doses (31). Although neostigmine is the most potent and preferred drug, high or unnecessary use was reported to increase post-operative respiratory morbidity (32-34). Therefore, monitoring BCHE polymorphisms in a population is important not only for the choice of relaxant but also for the consequences of blockade reversal.

In conclusion, the use of non-depolarizing relaxants that are not metabolized by acetylcholinesterase is not a definitive starting point for the prevention of postoperative residual curarization, as the effectiveness of drugs affecting the reversal of neuromuscular blockade is also affected by the presence or absence of genetic variants of cholinesterase. Screening for the occurrence of variants will be helpful for rapid and adequate treatment of postoperative residual curarization.
